# Molecular epidemiology, spatiotemporal patterns, and economic impact of African swine fever in Meghalaya, India

**DOI:** 10.3389/fcimb.2026.1889985

**Published:** 2026-08-12

**Authors:** Arockiasamy Arun Prince Milton, Kandhan Srinivas, Sabia Khan, Archana Thakur, Zakir Hussain, M. Saminathan, Sandeep Ghatak, G. Bhuvana Priya, Hosterson Kylla, Laureata Dkhar, Arnab Sen, Kekungu-u Puro, Samir Das

**Affiliations:** 1 Division of Animal and Fisheries Sciences, ICAR Research Complex for NEH Region, Umiam, Meghalaya, India; 2 Division of Virology, ICAR - Indian Veterinary Research Institute, Mukteswar, Uttarakhand, India; 3 College of Agriculture, Central Agricultural University (Imphal), Kyrdemkulai, Meghalaya, India; 4 Disease Investigation Office, Animal Husbandry and Veterinary Department, Shillong, Meghalaya, India

**Keywords:** African swine fever, *Asfivirus haemorrhagiae*, ASFV, economics, Meghalaya, outbreak

## Abstract

**Background:**

African swine fever (ASF), caused by *Asfivirus haemorrhagiae*, entered India through its northeastern frontiers in 2020. Following the initial outbreaks in Arunachal Pradesh and Assam, the next state to report ASF was Meghalaya, a tribal dwelling, pork-deficit state landlocked between Assam and the neighboring country of Bangladesh.

**Materials:**

In this study, we undertook an exploratory spatio-temporal analysis and economic cost analysis based on a total of 115 outbreak events involving 1603 animals were recorded involving 7 of the erstwhile 11 districts between July 2020 and April 2025. Additionally, we also regularly screened field samples from various districts of Meghalaya and tried to understand the molecular epidemiology based on *p72* and *p54* based phylogenetic analysis.

**Results and discussion:**

Phylogenetic analysis of representative strains from the outbreaks based on p72 and p54 regions assigned the strains to Genotype II, and more specifically sub-genotype IIa, respectively. Economic losses directly arising from assumed pork sales of affected animals yielded a conservative estimate of INR 2,74,45,883 (USD 305,225). Further, exploratory spatial autocorrelation analysis indicated clustering of outbreaks in 2020 and 2021. Moreover, exploratory temporal autocorrelation analysis hinted at weak but insignificant seasonal (p > 0.05) and annual trends at lag 12 frequency, which was also the feature observed in temporal decomposition of ASF outbreaks. Conclusions: The current study offers baseline data on which forecasting models can be developed and effectively prevent and contain future outbreaks by collaborative efforts of the stakeholders.

## 1 Introduction

African Swine Fever (ASF) is a highly contagious transboundary disease of viral etiology, affecting wild and domestic pigs, with case fatality rates reported up to 100%. ASF is also considered a notifiable disease, known for its history of significant negative socio-economic impacts, its ability to transgress across continents rapidly, and its potential to cause future harm in new geographical areas (WOAH, 2024). The etiological agent African swine fever virus (ASFV), also known as *Asfivirus haemorrhagiae*, is a double stranded DNA virus with a large envelope and highly complex antigenic structure. Additionally, the virus is the odd one among DNA viruses for its cytosolic replication and primarily affects macrophages and monocytes (Dixon et al., 2013). As the virus name suggests, the disease was first reported in the African continent a little more than a century ago in Kenya, following which the disease existed in endemic circulation in the porcine population of African countries. There are 24 known genotypes circulating in Africa and only genotype I and genotype II have transcended the continental boundaries to Portugal and Georgia in 1957 and 2007, respectively (Sanchez-Cordon et al., 2018). Though the initial 1957 event was brought under control, restricting the disease to the Italian island of Sardinia, the 2007 Georgian event triggered widespread dissemination of the virus to various countries of Europe and Asia. This took a dramatic turn in 2018 when incursion of ASF into China occurred, which paved way for further dissemination into India and the Southeast Asian countries (Rajukumar et al., 2021). Currently, ASF has reached the Latin American countries of Dominican Republic and Haiti in the Western hemisphere and Timor-Leste and Papua New Guinea in the Eastern hemisphere (Patil et al., 2020; Wang et al., 2023).

The epidemiology of the disease is considered to be complex, with multiple transmission cycles existing under field conditions. Transmission of the disease primarily occurs through direct contact with infected animals, contaminated feed and fomites. The virus can resist harsh environmental conditions and is known to persist in fomites and pork-associated products for a considerably long period (Chandana et al., 2024). Additionally, the transmission is also facilitated through soft ticks belonging to the genus *Ornithodoros* spp. in both domestic and wild pigs. Recently, mechanical transmission by bite of hematophagous insects such as *Stomoxys calcitrans* has also been shown to be possible (Olesen et al., 2018). The reservoir function of wild African suids such as red river hogs (*Potamochoerus porcus*), bushpigs (*Potamochoerus larvatus*), and warthogs (*Phacochoerus africanus*) might prove to be a major hindrance in controlling the disease in domestic pigs (Salguero, 2020). With the rising influence of climate change on various diseases, ASF in wild populations is vulnerable to fluctuations in climatic variables (Bergmann et al., 2021; Tiwari et al., 2022).

The disease manifests as a diverse spectrum of clinical signs in affected animals and is classified into peracute, acute, subacute and chronic forms (Li et al., 2022). Peracute form, often caused by highly virulent strain, kills the animal within 4 days, with associated signs including high fever, lethargy and anorexia (Patil et al., 2020; Bora et al., 2020, Cheng and Ward, 2022). The acute-form, being the most commonly featured during outbreaks, is characterized by high fever, hemorrhagic spots on the skin, bleeding from orifices, and pathology portraying multifocal hemorrhagic lymphadenitis and severe hemorrhagic splenomegaly (Patil et al., 2020). The sub-acute form is often caused by moderately virulent strains, and the clinical signs are similar to acute form but with reduced intensity. The chronic form is often a feature of the disease in endemic areas, with no clearcut vascular signs and often exhibits weight loss, respiratory distress, joint swelling and multifocal necrosis on the skin (Patil et al., 2020; Bora et al., 2020).

India houses around 9.06 million pigs and 46.85% are concentrated in the north-eastern states of India (Assam, Arunachal Pradesh, Meghalaya, Manipur, Nagaland Mizoram, Sikkim and Tripura), where pig farming plays an important role in supporting the livelihood of predominantly tribal populations (DAHD, 2019). India remained free of ASF until January 2020, when the first cases were reported in the states of Arunachal Pradesh and Assam with a history of sudden deaths in different villages along the river Brahmaputra (Rajukumar et al., 2021). Since then, the disease entered other neighboring states (Meghalaya in June 2020; Manipur in November 2020; Mizoram in March 2021; Nagaland in March 2021; Tripura in August 2021; and Sikkim in February 2022) and later disseminated to various parts of the country, severely hampering the pig and pork trade and resulting in tremendous economic implications (FAO, 2025; WAHIS, 2025).

The current study deals with the molecular epidemiology of ASF outbreaks in Meghalaya, a frontier state in Northeastern region of India sharing international boundaries with Bangladesh. The state is deficient in pig production and thus imports live animals from other states of the country to meet the meat demand of the population (Das et al., 2025a). We attempted an exploratory analysis to understand the spatial clustering and direct economic implications of ASF in the state from the first reported outbreak up to April 2025.

## 2 Methodology

### 2.1 Location and sample source

**Figure 1:**
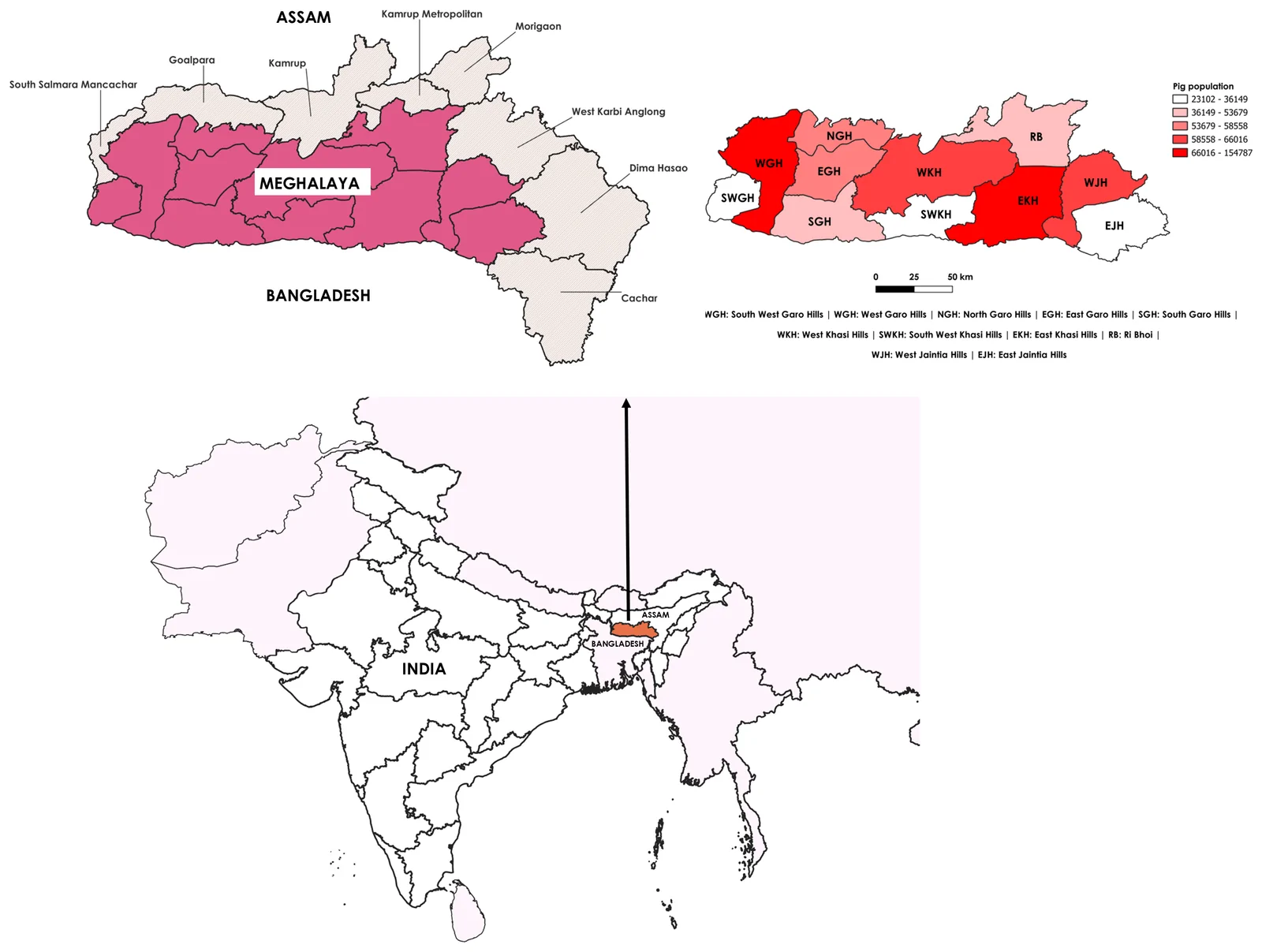
Demographic data on pig population of districts of Meghalaya considered for this study.

The study site is Meghalaya, which currently has twelve districts. The twelfth district, Eastern West Khasi Hills, was carved out of West Khasi Hills in 2021. We have still maintained the older administrative setup of eleven districts for ease of data analysis and availability of population data (**Figure 1**). Since the initial outbreak in the state in 2020, samples from animals suspected for ASF have been regularly submitted to the Division of Animal and Fisheries Sciences, ICAR Research Complex for NEH Region, for routine screening using World Organization for Animal Health (WOAH)-recommended diagnostic methods (WOAH, 2024). The ethical approval was obtained from the institute animal ethics committee (V-11011(13)/12/2023-CPCSEA-DADF).

### 2.2 Necropsy examination and sample collection

A field investigation team conducted post-mortem examinations and collected samples for molecular confirmation and histopathological studies. Samples were collected in sterile containers for molecular tests; whereas 10X neutral buffered formalin was used to store tissues meant for histopathology. A systematic necropsy examination was performed on dead pigs, inspecting external surfaces, body cavities, and internal organs for gross pathological lesions characteristic of ASF. Tissue samples from the trachea, lungs, heart, tonsils, lymph nodes, liver, spleen, intestine, kidneys, and blood samples were collected aseptically for further analysis, including histopathology and PCR.

### 2.3 Histopathology

Representatives tissue samples from affected areas were preserved in 10% neutral buffered formalin (NBF) for histopathological examination. Following adequate fixation in NBF, the tissues underwent standard processing for embedding in paraffin wax and were subsequently trimmed to produce sections of 4–5 μm thickness. These paraffin-embedded tissue sections were then stained using the conventional hematoxylin and eosin (H&E) staining method as described by Luna (1968) and were subsequently observed under a light microscope.

### 2.4 Sample processing and DNA extraction

The samples included tissue samples (liver, spleen and muscle) from dead animals, nasal swabs, blood and serum samples from animals in the surrounding surveillance zones. The samples were collected in sterile containers and transported to the laboratory at the earliest convenience possible for screening. Tissues from the same animal were pooled together and DNA extraction was carried out with DNeasy Blood& Tissue kit (Qiagen, Hilden, Germany) as per manufacturer’s protocol. The DNA concentration was estimated using a Nanodrop spectrophotometer (Thermo Scientific Fisher, Waltham, MA), and the extracted DNA obtained was subsequently stored at -20°C until further use.

### 2.5 PCR amplifications

Molecular confirmation involved screening the samples using TaqMan PCR-based assay as recommended by the WOAH (**Supplementary Table 1**), which is considered as the gold standard. The primers and the TaqMan probe were used in 20 pmol/µL and 10 pmol/µL concentrations, respectively. The 20 µL reaction mixture comprised of 10 µL of Luna^®^ qPCR Master Mix (New England Biolabs, Ipswich, USA), 1 µL of each primer, 0.2 µL of probe, 4.8 µL of nuclease-free water and 3 µL of template DNA. The cycling conditions used in the CFX Opus 96 real-time PCR instrument (Bio-Rad Laboratories, California, USA) comprised of 95 °C for 5 min, 45 cycles (95 °C for 15 s; 58 °C for 45 s) followed by hold. A Ct value of 40 was taken as the cut-off for differentiating positive from negative. To facilitate phylogenetic analysis, two additional regions, a 478 bp region of *B646L* gene (coding p72 protein) and a 683 bp region of the *E183L* gene (coding p54 protein) (**Supplementary Table 1**). The PCR conditions were followed as per published protocols. The reaction reagents involved 2X GreenTaq Mastermix (Thermo Fisher Scientific, Waltham, MA, USA) and oligonucleotide primers (M/S IDT, New Delhi, India). Amplification was performed using a Mastercycler^®^ Nexus X2 (Eppendorf, Hamburg, Germany). Post-PCR processing involved resolving the PCR products on a 1.5% agarose gel.

### 2.6 Phylogenetic analysis

Five representative samples from ASF outbreaks in Meghalaya were chosen for understanding the phylogenetic positioning. The specific bands corresponding to the desired amplicon sizes resulting from conventional PCR were excised, purified and outsourced to Eurofins Genomics India Ltd. for Sanger sequencing. The sequencing reads were checked for quality and trimmed using BioEdit v.7.7.1 (Hall, 1999). The curated reads were assembled using MEGA v12.0.9 (Kumar et al., 2024) and EMBOSS CONS (Madeira et al., 2024; available at https://www.ebi.ac.uk/jdispatcher/msa/emboss_cons). The assembled reads were verified with blastn tool of NCBI and submitted to NCBI BankIt for obtaining accession numbers. For phylogenetic analysis based on *B646L* gene, sequences from a global dataset were retrieved to include all known 24 genotypes. For the *E183L* gene, representative sequences were selected to include all known *E183L* (*p54*)-based subtypes of Genotype II prevalent in the neighboring regions. Multiple sequence alignment was facilitated using NCBI BLAST and MUSCLE algorithm of MEGA. The in-built model selection tool identified Tamura 3-parameter model (Tamura, 1992) and Kimura 2-parameter model (Kimura, 1980) as the best-fit models for *B646L* and *E183L* gene-based alignments, respectively, to construct maximum likelihood trees with 1000 bootstrap replicates (Felsenstein, 1985).

### 2.7 Data collection on ASF outbreaks in Meghalaya

Data on ASF outbreaks throughout the state from the first reported outbreak up to the cut-off date of April 15, 2025, were collected from the Disease Investigation Office of Animal Husbandry and Veterinary department of Meghalaya. The data included the timelines of outbreaks, village/epicenters and the number of animals affected. District-wise mortality rates were calculated using total susceptible animals derived from the last livestock census, present in the affected villages, as the denominator and multiplied by 100 (**Table 1**).

**Table 1:** Results of laboratory-based ASF Screening conducted during the period of study with details on yearly outbreaks reported with annual mortality rates in each district.

Year	District	Samples tested	PCR positive (%)	No. of villages affected/epicenters reported	No. of animals affected/no. of susceptible animals*	Mortality rate (percent)
2020	RB	10	7(70.00)	11	113/970	11.65
	EKH	16	6(37.50)	12	19/868	2.19
	WKH	NT	NT	3	7/505	1.39
	SWKH	NT	NT	1	5/590	0.85
	EJH	1	0 (0.00)	2	6/167	3.59
	WJH	17	6 (35.29)	5	47/486	9.67
Annual total		44	19 (43.18)	34	197/3586	5.49
2021	RB	5	2 (40.00)	11	98/1328	7.38
	EKH	7	3 (42.86)	14	117/2685	4.36
	WKH	17	5 (29.41)	1	85/468	18.16
	SWKH	NT	NT	3	37/209	17.70
	WJH	NT	NT	1	1/975	0.10
Annual total		29	10 (34.48)	31	338/5665	5.97
2022	RB	42	13 (30.95)	9	588/1496	39.31
	EKH	30	4 (13.33)	1	1/160	0.63
Annual total		72	17 (23.61)	10	589/1656	35.57
2023	RB	25	11(44.00)	19	174/1782	9.76
	EKH	10	7 (70.00)	4	8/385	2.08
	WKH	1	1 (100.00)	4	49/534	9.18
	WJH	4	0 (0.00)	0	NA	0.00
	SGH	1	1 (100.00)	1	7/20	35.00
	WGH	1	1 (100.00)	3	149/227	65.64
Annual total		42	21 (50.00)	31	387/2948	13.13
2024	RB	7	3 (42.86)	2	2/164	1.22
	EKH	15	5 (33.33)	5	79/284	27.82
	SWKH	1	1 (100.00)	1	1/40	2.50
	WKH	NT	NT	1	2/450	0.44
Annual total		23	9 (39.13)	9	84/938	8.96
2025	EKH	8	1 (12.50)	1	1/25	4.00
	WJH	4	1 (25.00)	1	7/24	29.17
	RB	5	0 (0.00)	0	NA	0.00
Annual total		17	2 (11.76)	2	8/49	16.33
Overall		227	78 (34.36)	115	1603/14842	10.80

* The total number of susceptible animals in each affected village was considered for calculating the mortality rate for each district.

### 2.8 Economic loss approximation from the ASF outbreaks

For economic cost estimations, information on the average yield per animal data in each district was collected from the annual Integrated Sample Survey records of Animal Husbandry and Veterinary department (Government of Meghalaya, 2025). Market prices of pork for the previous years were obtained through personal enquiries with stakeholders.

### 2.9 Exploratory spatial data analysis

Exploratory spatial autocorrelation analysis was conducted using Global Moran’s I Index (Moran, 1950), local indicators of spatial association (LISA) (Anselin, 1995; 2019) and Getis Ord-Gi* statistics (Ord and Getis, 2001; Getis and Ord, 2010). The open source GeoDA v1.22.0.21 software (Anselin et al., 2022) was used for analysis. The spatial patterns associated with ASF outbreaks and animal mortality were treated as events and compared longitudinally across years. Temporal analysis was also undertaken to better understand outbreak patterns and disease intensity. As data for 2025 was incomplete, spatial and temporal year-wise comparisons were restricted to data up to 2024. A spatial weight matrix was generated using a queen contiguity matrix, which considers each district that shares an edge or vertex as neighbors to compute the spatial parameters (Anselin, 1988). Global Moran’s *I* value ranged from negative to positive unity; with values away from zero (randomness) towards positive side indicated clustering and towards negative side indicated dispersion (Moran, 1950). For statistical rigor, computations were maintained at 99999 permutations to generate I, Z and pseudo p values. Spatial autocorrelation was considered significant when the Z score exceeded ±1.96, corresponding to a two-tailed significance level of p < 0.05. LISA identifies spatial clusters and outliers and categorizes districts into high-high, low-low, low-high and high-low clusters *i.e.* indicating the pattern of events in relation to the neighbors (Anselin, 1995; 2019). To identify potential hotspots or coldspots of the disease and outbreak occurrence, Getis-Ord-Gi* statistic with row-standardized weights was computed for aggregated counts and yearly counts (Ord and Getis, 2001; Getis and Ord, 2010). District-wise population data from 20^th^ Livestock census were used wherever necessary (DAHD, 2019) (**Figure 1**).

### 2.10 Exploratory temporal trend analysis

Outbreak data across 5 years were subjected to exploratory temporal trend analysis to assess autocorrelation and seasonal variation. The months from November to February were considered as winter; March to June as Summer; and July to October as monsoon for comparison purposes (Government of Meghalaya, 2025). Kruskal Wallis test was employed to compare the number of outbreaks across the three seasons. Autocorrelation function (ACF) and partial autocorrelation function (PACF) (Box et al., 2015) with Barlett approximation (Barlett, 1946) and Ljung-Box Q test (Ljung and Box, 1980) were used to assess annual trends at a lag frequency of 12. Augmented Dickey-Fuller test was used to assess stationarity (Said and Dickey, 1984). Additionally, temporal decomposition was performed using seasonal-trend decomposition using Locally Estimated Scatterplot Smoothing (LOESS) method (Cleveland et al., 1990). The exploratory temporal trend analysis was performed using RStudio v2025.09.1 (R v.4.4.1) using packages “forecast” (v9.0.0; Hyndman et al., 2026), “astsa” (v2.4; Stoffer, 2025) and “tseries” (v0.10-59; Trapletti and Hornik, 2026).

## 3 Results

### 3.1 Screening of samples

Various clinical samples belonging to 231 animals were received during the study period from various districts such as West Khasi Hills, East Khasi Hills, South West Khasi Hills, South Garo Hills, East Jaintia Hills, West Jaintia Hills and Ri Bhoi. The district-wise sample and positivity details are provided in **Table 1**. The samples which gave amplification peaks in TaqMan PCR below Ct value of 40 were considered as positive. Overall positivity of ASF in the samples tested as part of routine screening was 34.36% (95% Wilson’s confidence interval: 28.49% - 40.75%). The positivity rates were highest in 2023 (50.00%), followed by 2020 (43.18%), 2024 (39.13%), 2022 (34.48%), 2022 (23.61%) and 2025 (11.76%). On aggregating the samples received for the entire study period, the positivity rates were 100% for South Garo Hills, West Garo Hills, South West Khasi Hills, but only one sample was screened from each district. East Jaintia Hills was another district where only one sample was screened, which yielded a negative result. Among the remaining districts, highest positivity rate was observed for Ri Bhoi (38.29%), followed by East Khasi Hills (30.23%), West Khasi Hills (33.33%) and West Jaintia Hills (2.80%) (**Table 1**).

### 3.2 Pathomorphological features of ASF outbreaks

**Figure 2:**
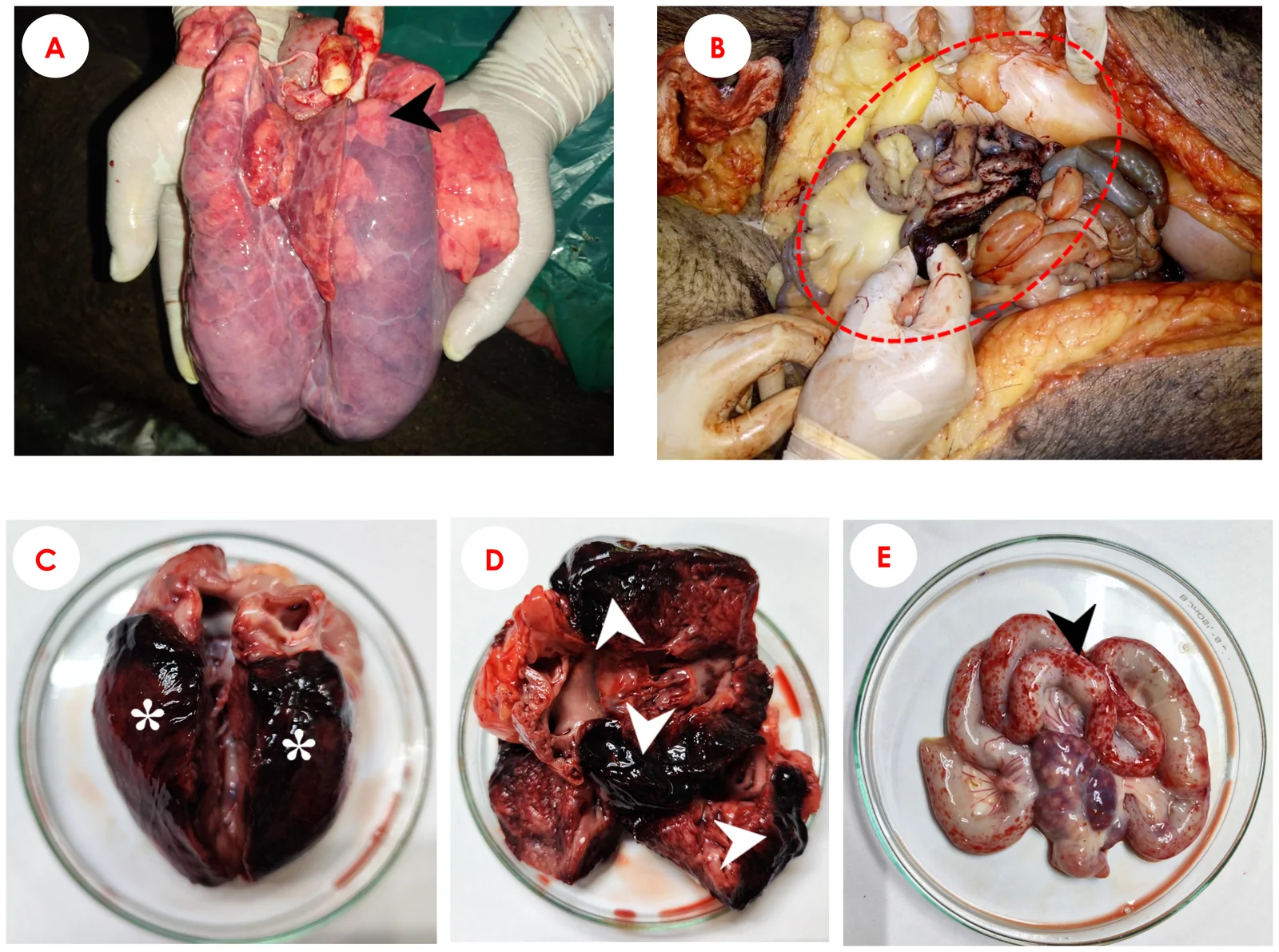
Gross pathological lesions observed in outbreaks of African swine fever. **(A)** Lungs showed marbling appearance (arrowhead), edema, congestion, and enlargement in size. **(B)** Small intestinal serosal surface showed marked petechial and ecchymotic hemorrhages. **(C)** Epicardial surface showed diffuse severe hemorrhages (asterisk). **(D)** Endocardial surface showed diffuse severe hemorrhages (arrowhead). **(E)** Small intestinal serosal surface showed paint-brush hemorrhages (arrowhead).

**Figure 3:**
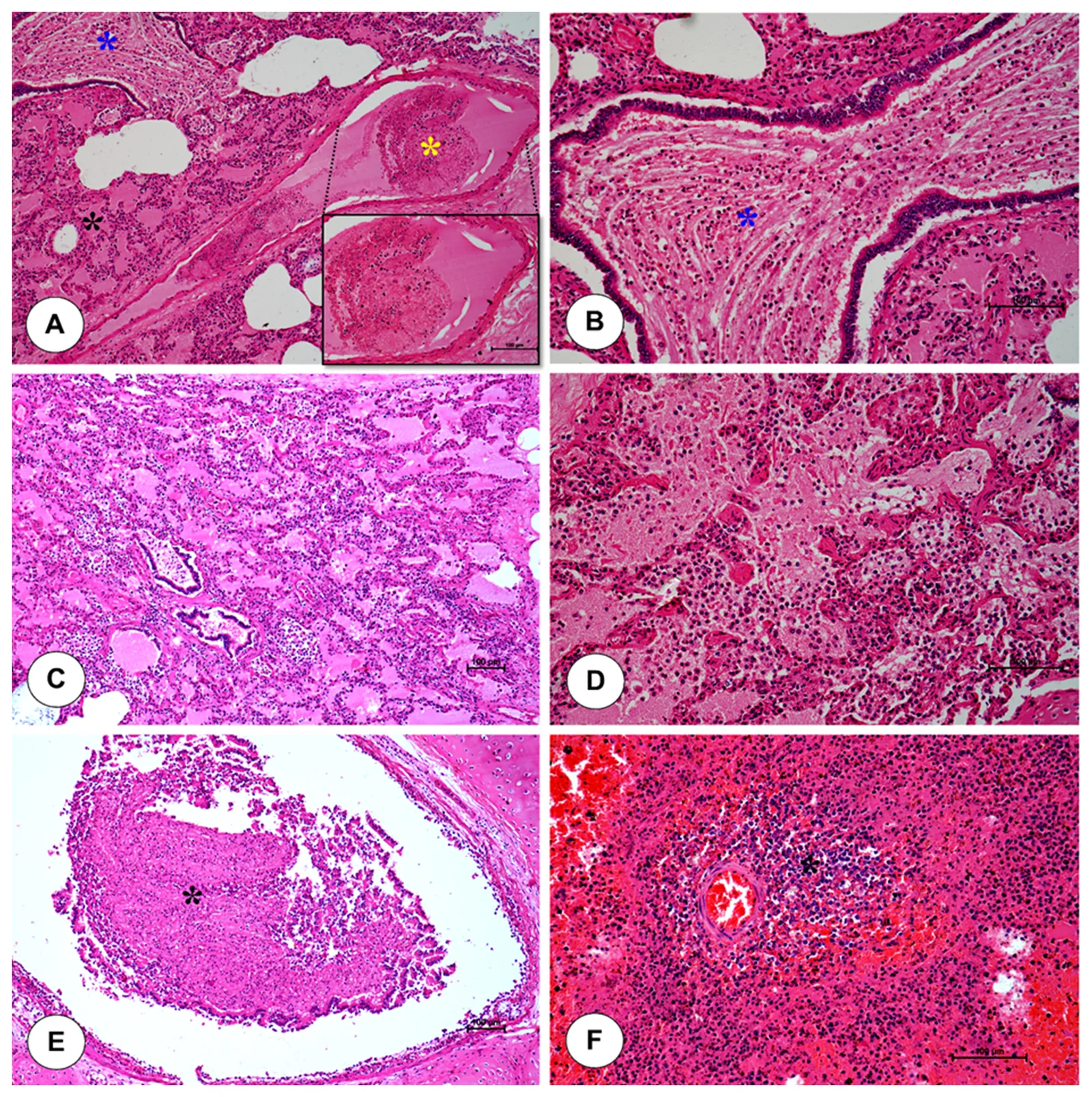
Histopathological lesions in pigs naturally infected with African swine fever virus. **(A)** Lungs showed severe pulmonary edema, alveoli filled with protein-rich eosinophilic edematous fluid (*) admixed with few inflammatory cells and emphysematous alveoli. Bronchiole filled with fibrin (*) admixed with inflammatory cells. Blood vessel showed thrombi attached with blood vessel wall (*). H&E x100. Inset, Higher magnification of thrombi. H&E x200. **(B)** Higher magnification of panel **(A)** Bronchiolectasis with dilatation of bronchiole filled with fibrin and admixed with marked inflammatory cells. H&E x200. **(C)** Severe pulmonary edema and alveoli filled with edematous fluid admixed with marked inflammatory cells. H&E x100. **(D)** Higher magnification of Panel **(C)** Ruptured alveoli filled with edematous fluid admixed with marked inflammatory cells and fibrin. H&E x200. **(E)** Bronchiectasis with dilated affected bronchi and lumen filled with desquamated epithelial cells, inflammatory cells and fibrin (*). H&E x100. **(F)** Spleen showed marked lymphoid depletion in white pulp (*) due to lymphocytolysis. H&E x200.

**Figure 4:**
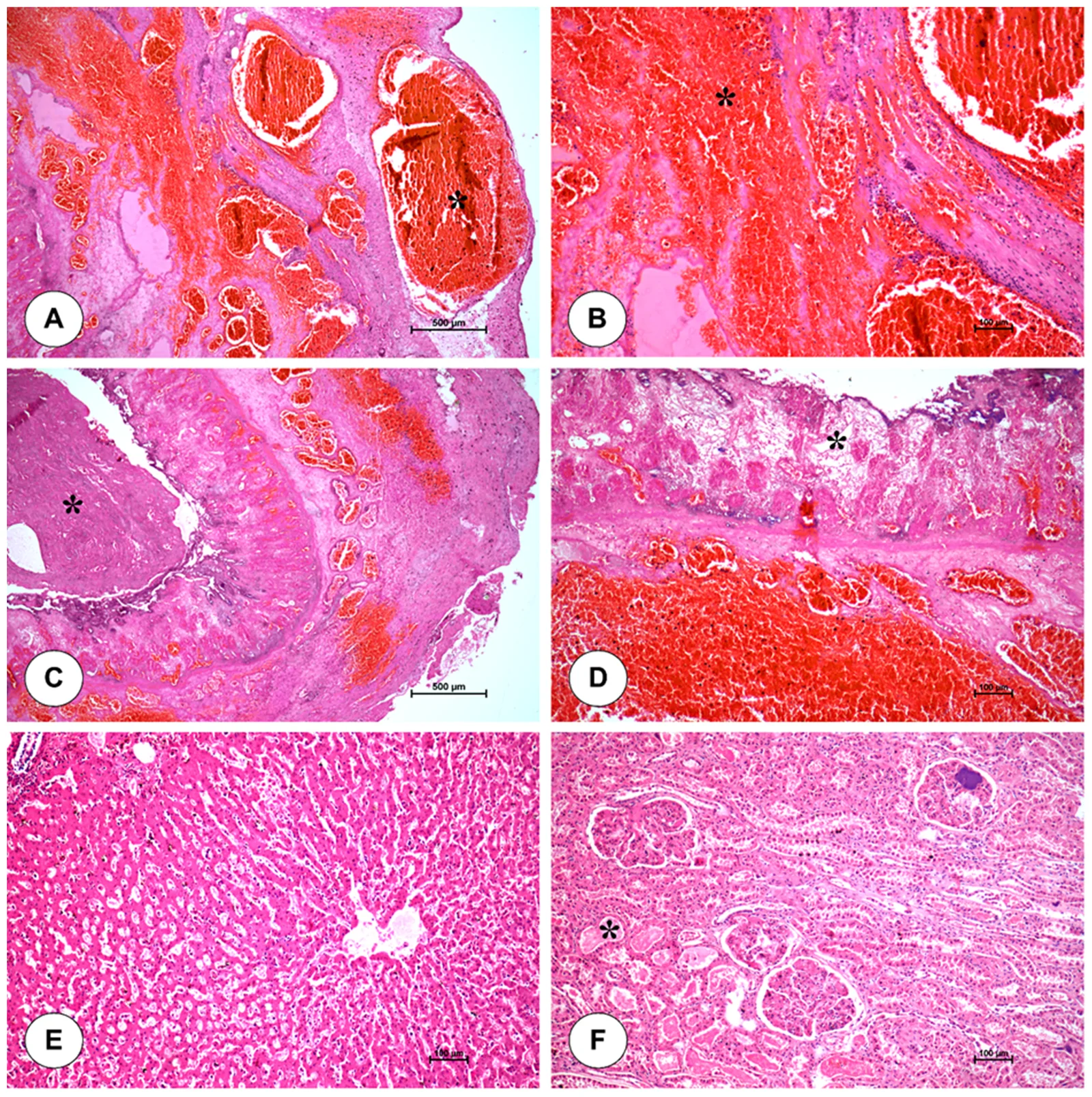
Histopathological lesions in pigs naturally infected with African swine fever virus. **(A)** Acute, extensive, severe, necro-hemorrhagic enteritis. Severe acute coagulative necrosis, hemorrhages, edema, and congestion of blood vessels (*) in serosa, muscularis externa, and submucosa of intestine. H&E x40. **(B)** Higher magnification of Figure **(A)** Severe necro-hemorrhagic enteritis with massive hemorrhages (*). H&E x200. **(C)** Severe necro-hemorrhagic enteritis and intestinal lumen contained fibrino-coagulated necrotic cellular debris with denuded necrotic epithelial cells, inflammatory cells and fibrin (*). H&E x40. **(D)** Higher magnification of Panel **(C)** Severe necro-hemorrhagic enteritis with massive coagulative necrosis of intestinal villi (*). H&E x200. **(E)** Centrilobular hepatocyte atrophy. Liver showed dilated sinusoidal spaces with atrophy of hepatocytes. H&E x200. **(F)** Kidneys showed dilated tubules with protein-rich eosinophilic fluid (*), degeneration of tubular epithelial cells and colony of bacteria in Glomeruli. H&E x100.

Animals showed weakness, dullness, lethargy and huddling. Other clinical features included high fever, inappetence, abortions in pregnant sows and sudden death. Salient gross pathological observations included splenomegaly with hemorrhages and infarctions, paintbrush like hemorrhages in the intestinal serosa, hemorrhages in the pericardium and lungs, and kidneys with petechial hemorrhages resembling a turkey egg (**Figure 2**). The histopathological lesions in pigs naturally infected with African swine fever virus included severe pulmonary edema, alveoli filled with protein-rich eosinophilic edematous fluid admixed with marked inflammatory cells and emphysematous alveoli in lungs (**Figure 3A, C**). Bronchiolectasis with dilatated bronchioles filled with fibrin and inflammatory cells was observed (**Figure 3B**). Bronchiectasis with dilated bronchi and lumen filled with desquamated epithelial cells, inflammatory cells and fibrin were noticed (**Figure 3E**). Ruptured alveoli filled with edematous fluid admixed with inflammatory cells and fibrin were observed (**Figure 3D**). Blood vessels showed thrombi attached to the blood vessel wall (**Figure 3A**). Spleen showed marked lymphoid depletion in white pulp due to lymphocytolysis (**Figure 3F**). Intestine showed acute, extensive, severe, necro-hemorrhagic enteritis with coagulative necrosis, massive hemorrhages, edema, and congestion of blood vessels (**Figures 4A–D**). Intestinal lumen contained fibrino-coagulated necrotic cellular debris with denuded necrotic epithelial cells, inflammatory cells and fibrin (**Figure 4C**). Massive coagulative necrosis of intestinal villi was noticed (**Figure 4D**). Liver showed centrilobular hepatocyte atrophy and dilated sinusoidal spaces (**Figure 4E**). Kidneys showed dilated tubules with protein-rich eosinophilic fluid, degeneration of tubular epithelial cells and bacterial colonies in glomeruli (**Figure 4F**).

### 3.3 Phylogenetic analysis

**Figure 5:**
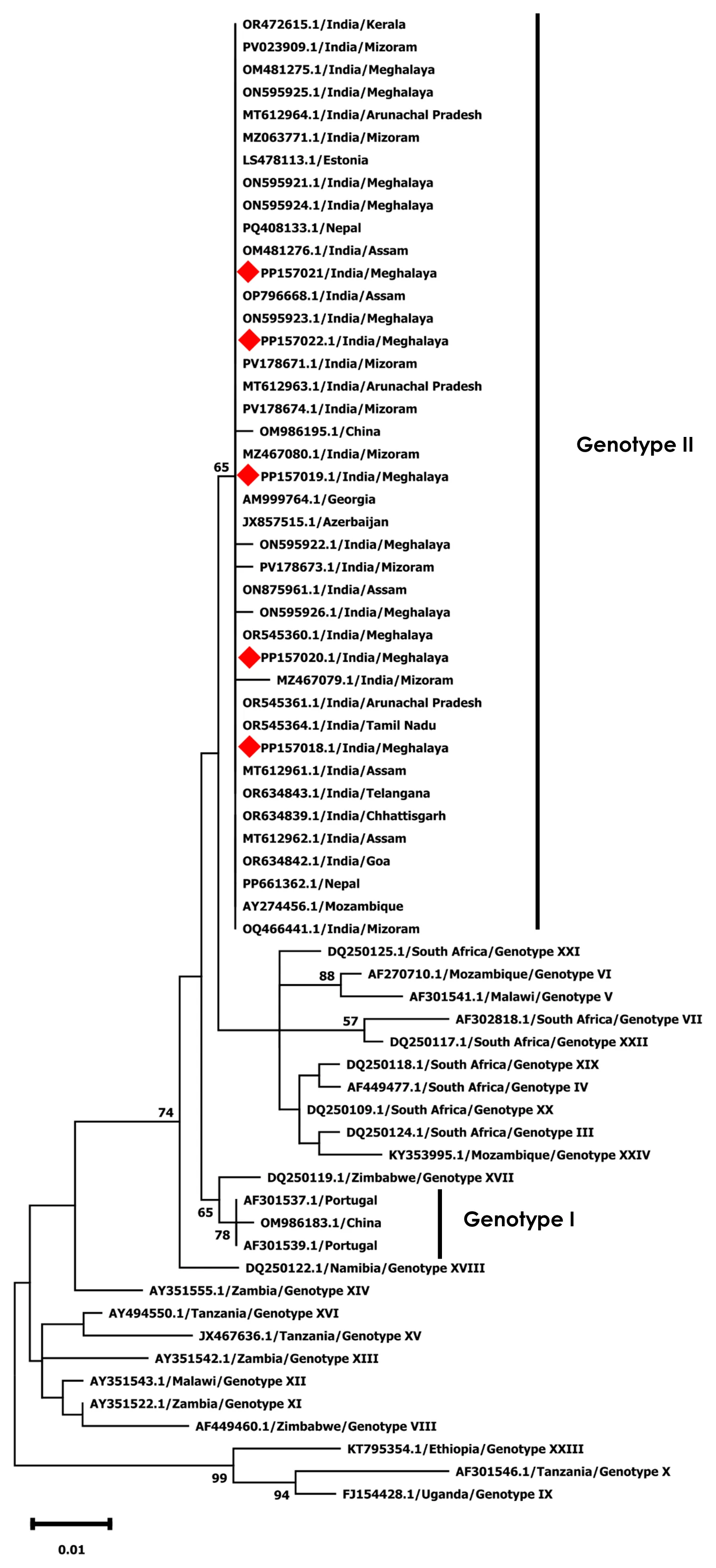
Maximum likelihood tree based on *p72* gene placing the outbreak strains in genotype II. The sequences generated in this study are indicated by a red marker.

**Figure 6:**
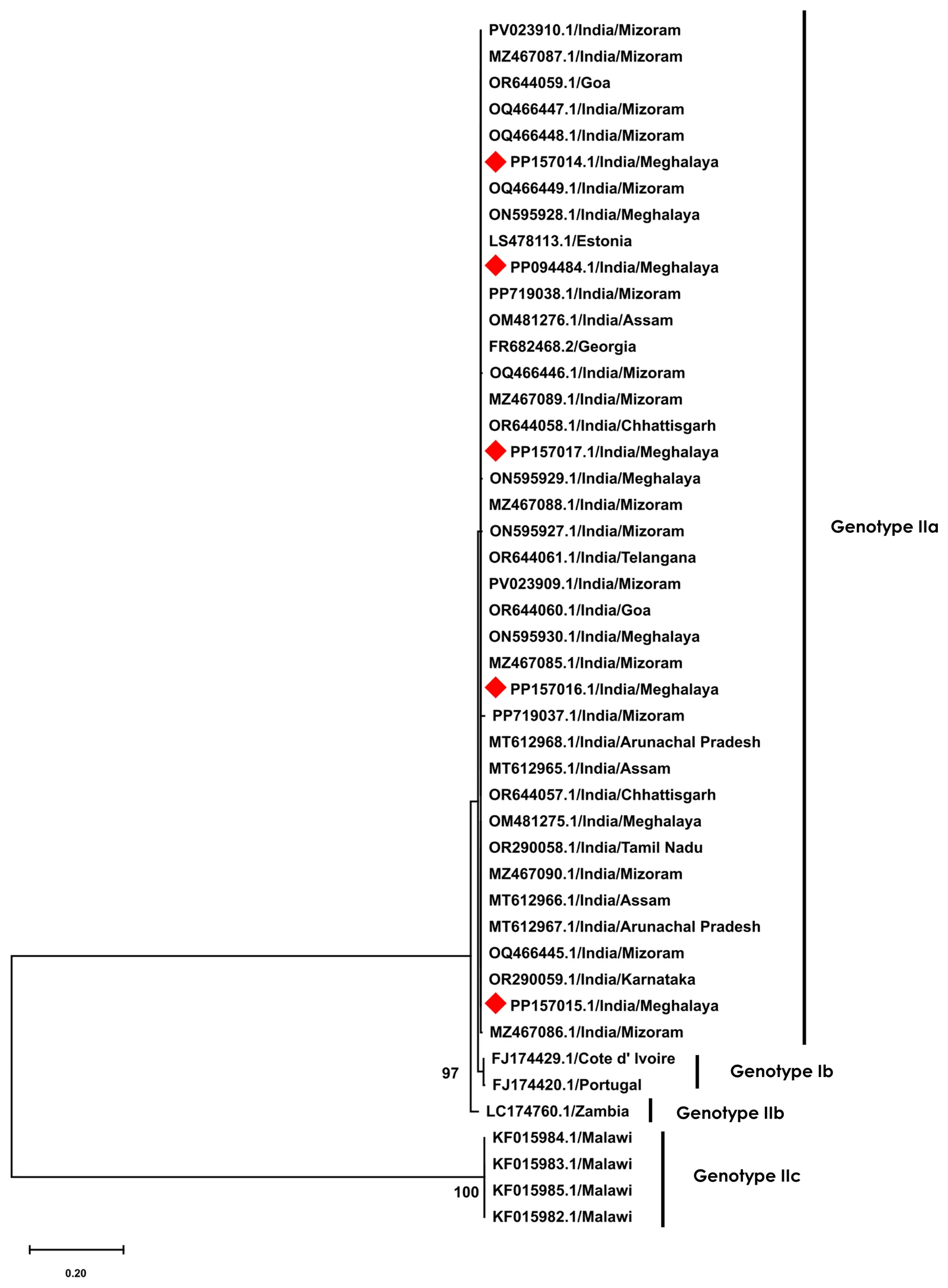
Maximum likelihood tree based on p54 gene placing the outbreak strains in genotype IIa. The sequences generated in this study are indicated by a red marker.

Bi-directional Sanger sequencing of the *B646L* and *E183L* region-based amplicons further confirmed the diagnosis of ASF. The sequences showed 100% identity with previously reported sequences from India as well as other countries such as Ghana, Korea, Lithuania, and Papua New Guinea. The accession numbers for the assembled *B646L* regions (PP157018, PP157019, PP157020, PP157021, PP157022) and *p54* regions (PP094484, PP157014, PP157015, PP157016, PP157017) corresponding to each sample were obtained from NCBI after successful submission. Phylogenetic analysis based on the amplified *B646L* region using a maximum likelihood algorithm and reference strains representing all 24 genotypes, revealed that all the strains sequenced in this study belonged to Genotype II of ASFV (**Figure 5**). Similarly, *p54*-based phylogenetic analysis placed the strains in sub-genotype IIa, clustering closely with strains reported from the Indian states of Mizoram, Meghalaya, Chhattisgarh, Karnataka in addition to Georgia and Estonia (**Figure 6**).

### 3.4 Outbreaks

**Figure 7:**
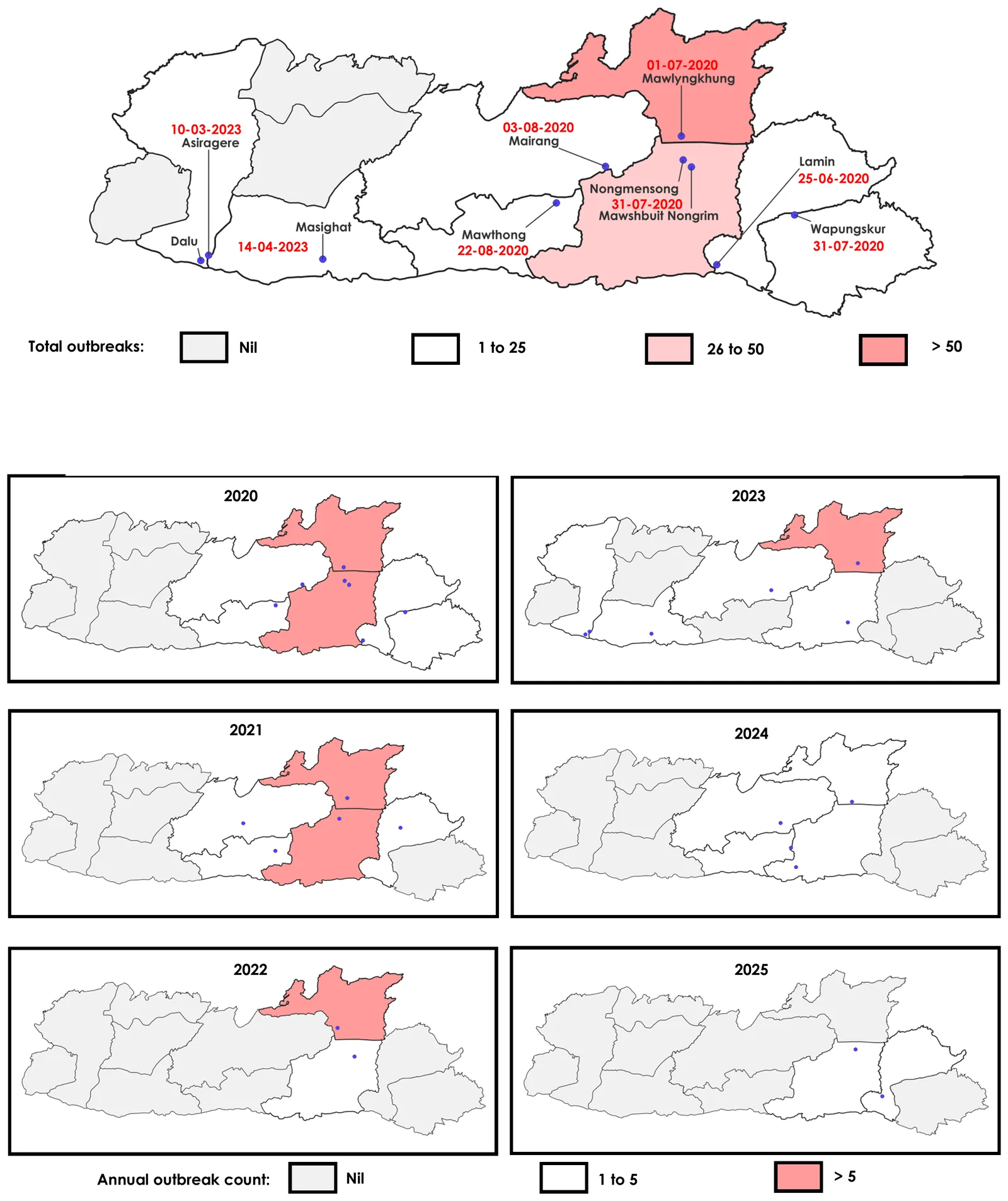
Timeline of first outbreaks in various spatial units in each year.

First outbreak of ASF in Meghalaya occurred in Lamin village of the Amlarem sub-division of West Jaintia Hills on 25^th^ June 2020 (**Figure 7**, Supplementary Table 2). Since then, a total of 115 outbreak events were reported up to 15 April 2025, affecting approximately 1603 animals. Following the Lamin outbreak, the next district to be affected was Ri Bhoi in the first week of July 2020. By the end of July, the disease was simultaneously reported East Khasi Hills and East Jaintia Hills in Nongmensong (East Khasi Hills), Mawshbuit Nongrim (East Khasi Hills) and Wapungskur (East Jaintia Hills), respectively (**Figure 7**, **Supplementary Table 2**). Two more districts, West Khasi Hills and South West Khasi Hills were affected in August 2020, with outbreaks reported in Mairang and Mawthong villages, respectively. West Garo Hills and South Garo Hills remained naïve to ASF until 2023, when outbreaks were reported from Dalu, Asiragre and Masighat villages (**Figure 7**, **Supplementary Table 2**).

In the study period, maximum outbreak events were reported from Ri Bhoi (52) followed by East Khasi Hills (36), West Khasi Hills (11), West Jaintia Hills (5), South West Khasi Hills (5), West Garo Hills (3), East Jaintia Hills (2) and South Garo Hills (1) (**Table 1**). While considering the number of animals affected and died due to ASF, Ri Bhoi was the most affected district (975), followed by East Khasi Hills (225), West Garo Hills (149), West Khasi Hills (143), West Jaintia Hills (55), South West Khasi Hills (49), South Garo Hills (7) and East Jaintia Hills (6). Single outbreak with the highest mortality was in a government regional pig breeding farm at Kyrdemkulai, Ri Bhoi, in 2022, where 530 deaths were recorded. Over the years, the highest number of outbreak events was recorded in 2020 (34), followed by 2023 (31), 2010 (30), 2022 (10), 2024 (9) and 2025 (2). The highest mortality rate was observed in West Garo Hills (65.64%), which was also the highest value for a single year (2023). It was followed by South Garo Hills (35.00%), Ri Bhoi (16.99%), West Khasi Hills (7.31%), South West Khasi Hills (5.13%), East Khasi Hills (5.11%), West Jaintia Hills (3.70%) and East Jaintia Hills (3.59%) (**Table 1**).

### 3.5 Economics

The total direct economic loss in terms of pork production arising from ASF outbreaks amounted to a conservative estimate of INR 2,74,45,883, equivalent to USD 305,225 (1 USD = INR 89.92). Losses were proportional to the number of cases reported annually, with the highest loss recorded in 2022 (USD 112,709), followed by 2023 (USD 80,967), 2021 (USD 58,642), 2020 (USD 32,168), 2024 (USD 18,860) and 2025 (USD 1,878).

### 3.6 Spatial autocorrelation analysis

Global Moran’s *I* value indicated randomness of the ASF cases when aggregated over 5 years (**Table 2**). Statistically significant clustering was observed in 2020 (I value: 0.222; Z value: 2.30; p value: 0.03) and 2021 (I value: 0.344; Z value: 2.05; p value: 0.04). Aggregated outbreak data showed significant spatial clustering (I value: 0.259; Z value: 1.97; p value: 0.04). Year-wise, significant clustering of outbreaks was observed only in 2020 (I value: 0.444; Z value: 2.66; p value: 0.04). Outbreak clustering in 2021 was borderline (I value: 0.248; Z value: 1.83; p value: 0.04) (**Table 2**).

**Table 2:** Global Moran’s I statistics obtained from Spatial autocorrelation analysis.

Year	*I* value	Z value	Pseudo p value	Clustering?
Aggregated cases	0.031	1.23	0.10	No
2020	0.222	2.30	0.03	Yes
2021	0.344	2.04	0.04	Yes
2022	(-) 0.074	0.50	0.37	No
2023	(-) 0.287	(-) 0.95	0.13	No
2024	(-) 0.096	0.06	0.48	No
Aggregated outbreaks	0.259	1.97	0.04	Yes
2020	0.444	2.66	0.02	Yes
2021	0.248	1.83	0.04	Yes
2022	(-) 0.027	1.15	0.07	No
2023	0.003	0.92	0.16	No
2024	0.184	1.93	0.04	Borderline

LISA identified high-high clustering pattern in East Khasi Hills for the aggregated cases data, bordered by West Jaintia Hills with a low-high clustering pattern (p < 0.05). On the other side of Meghalaya, West Garo hills exhibited high-low clustering pattern (p<0.05). In the year 2020, when the first outbreak was reported, Ri Bhoi, East Khasi Hills and West Jaintia hills showed high-high clustering (p < 0.05) (**Supplementary Figure 1**). Ri Bhoi and East Khasi Hills districts also exhibited a high-high clustering pattern in 2021. The clustering pattern moved towards insignificance levels in the years 2022 and 2023. Only high-low and low-high patterns were noticed in the year 2024. Similar pattern was also noticed when considering the number of outbreaks over the years. On aggregated data, Ri Bhoi and East Khasi Hills exhibited high-high clustering (p < 0.05) (**Supplementary Figure 1**). In the year of first outbreak, a high-high pattern was observed in West Jaintia Hills in addition to the previous year. The gradual trend towards insignificance and less clustering was also noticed in 2022 to 2023. In the year 2024, East Khasi Hills exhibited a high-high clustering pattern bordered by West Jaintia hills which exhibited a low outbreak count surrounded by districts with high outbreak counts (p < 0.05) (**Supplementary Figure 1**).

Getis-Ord Gi* statistics was used to indicate statistically significant coldspots and hotspots of ASF cases and outbreaks (**Supplementary Figure 2**). On analysis of the aggregated case data, East Khasi Hills and West Jaintia Hills were identified as hotspots, whereas West Garo Hills was identified as a cold spot. This trend was also observed when outbreaks were considered instead of cases. In 2020 and 2021, Ri Bhoi and East Khasi Hills served as the hotspots for both ASF cases and outbreaks, while West Garo Hills exhibited as coldspot in four out of five years (**Supplementary Figure 2**).

### 3.7 Temporal autocorrelation and annual trend analysis

**Figure 8:**
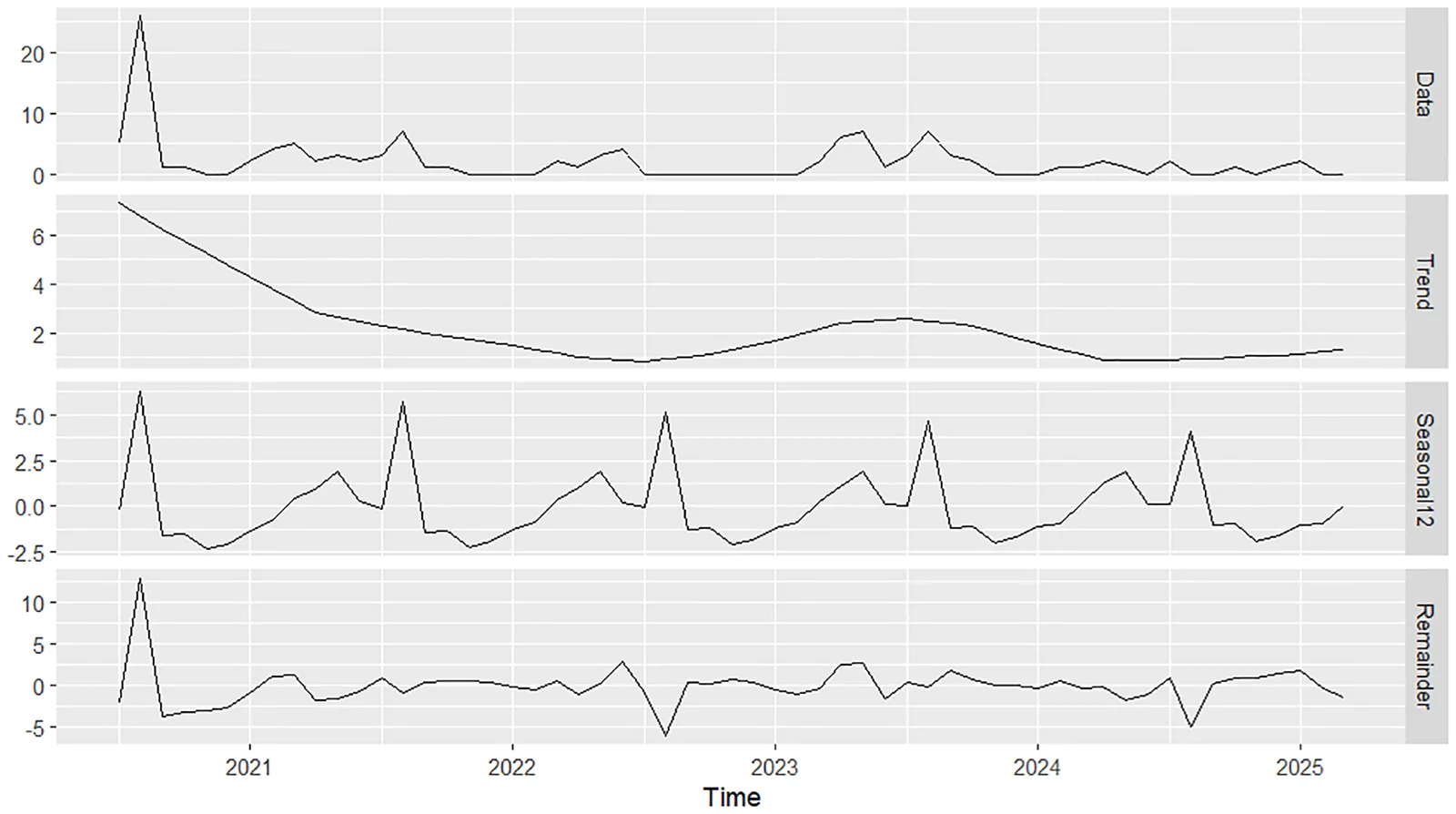
Time-series decomposition of monthly outbreaks of ASF in the study period.

Exploratory temporal analysis did not identify any significant seasonality in outbreaks. Even though ASF outbreaks were found to occur apparently more frequently in the monsoon season (n = 63) when compared to summer (n = 42) and winter (n = 10) the apparent seasonal variations were not found to be statistically significant across the years (p > 0.05). Autocorrelation function analysis indicated a weak positive correlation at a 12-month lag (autocorrelation = 0.165; SE = 0.139; ADF = -4.0795; p value = 0.01249) (**Supplementary Figure 3**). However, Ljung-Box test indicated no statistically significant autocorrelation up to 12 months (Ljung Box Q [12] = 5.09; and p = 0.955). PACF analysis (autocorrelation = 0.143; SE = 0.132) also supported the lack of evidence of direct annual (12-month) dependence in the monthly outbreaks (**Supplementary Figure 3**). STL decomposition analysis revealed a gradually decreasing trend since the initial peak observed in 2020; with a minor peaking noticed around 2023. Seasonality, though observed, was considered weak, and the residuals did not follow any observable pattern (**Figure 8**).

## 4 Discussion

The transgression of ASF into Indian territory started a public health scare in terms of food security, loss of livelihood and other economic implications. The effects were more strongly felt in areas where pig rearing was the primary livelihood occupation. The current study focuses on the occurrence, diagnosis, economic costs, spatial and temporal trends of ASF in the tribal state of Meghalaya. Meghalaya is present in the northeastern region of India, which is characterized by porous international borders, where unauthorized cross-border movement of people and livestock is not uncommon. The routine laboratory diagnostic screening efforts undertaken since the first outbreak noticed in the state of Meghalaya in July 2020 assisted in keeping tabs on the disease by providing timely diagnosis. Excluding districts from where only one samples were tested, the highest rates were noticed in Ri Bhoi Hills and East Khasi Hills (**Table 1**). The hilly terrain of the state often impedes the transportation of samples for routine diagnosis and might have contributed to the low number of samples screened. Though the overall positivity rate (34.36%) falls between 16.72%, as reported in a cross-sectional study from the neighboring state of Assam (Choudhury et al., 2026), and 68.03% reported from a multi-state study in India (Sai Balaji, 2023); the rates could not be effectively compared, as the current study spans an approximately 5-year period. The positivity rates might have also varied with the choice of diagnostic methods with improved sensitivity such as isothermal amplification methods, nested PCR and real-time PCR (Milton et al., 2023a, 2024). Moreover, the positivity rates might have varied due to probable sample biasness arising due to testing of naturally infected population in the study area.

The clinical signs and history observed in outbreaks of Meghalaya were similar to those described in other studies reporting an acute manifestation of ASF in naïve population (Marak et al., 2022; Pegu et al., 2023; Gupta et al., 2024; Narnaware et al., 2025; Verma et al., 2024). Acute manifestations are usually the result of infection with highly virulent or moderately virulent strains of ASFV (Salguero, 2020). Gross lesions such as splenomegaly and hemorrhagic lesions on visceral organs also indicated an acute form of ASF, as reported earlier (Pegu et al., 2023; Narnaware et al., 2025). The pathology of ASF is characterized by widespread hemorrhages, necrosis, and inflammation in multiple organs. The acute form is often fatal due to massive hemorrhagic lesions and organ failure. In the present study, severe hemorrhagic lesions were suggestive of the acute lethal form of ASF (Pikalo et al., 2019). Acute, highly virulent infection with ASFV typically results in severe hemorrhagic lesions, widespread organ involvement, and rapid death. Congestion, hemorrhages, and tissue necrosis observed in the present study were characteristic lesions observed during the early stage of infection (Pikalo et al., 2019). In the present study, pathological lesions observed in the infected pigs like pulmonary hemorrhages and edema indicative of extensive endothelial damage caused by the virus, were consistent with earlier reports (Ruedas-Torres et al., 2024).

Liver lesions in ASF-infected pigs were in agreement with the previous findings of pathological changes, including hepatitis, necrosis, congestion, hemorrhage, and biliary tract involvement (Sehl-Ewert et al., 2022). In the present study, kidneys displayed tubular degeneration and necrotic changes with sloughing of epithelial cells, which were consistent with the previous studies (Hervás et al., 1996). Studies have reported that ASFV replication in mesangial cells and renal collecting duct epithelial cells may lead to the presence of the virus in urine, even in the absence hematuria (Hervas et al., 1996). ASFV infection often leads to severe hemorrhagic lesions in the spleen, which are hallmark of ASF.

Lymphoid depletion in the spleen, affecting both white pulp (lymphoid follicles) and red pulp and disrupting the normal splenic architecture, contributes to immune suppression and increased susceptibility to secondary infections in highly virulent infections (Carrasco et al., 1997). ASF can cause various lesions in intestine, although they are generally less pronounced compared to lesions in other organs such as the spleen and lymph nodes. Common findings include congestion, hemorrhages, and necrosis of the intestinal mucosa. In the present study, intestine exhibited hemorrhage and necrosis of the mucosa and submucosa. These findings reflect the acute and often severe nature of ASF infection (Salguero, 2020).

Phylogenetic analysis based on *B646L* (*p72*) gene placed the isolates from Meghalaya in Genotype II, which is also the only reported genotype in India till now (Rajukumar et al., 2021; Marak et al., 2022; Rajkhowa et al., 2022; Buragohain et al., 2023; Hiremath et al., 2024; Narnaware et al., 2025). Additionally, phylogenetic proximity with strains reported from other countries such as Estonia, Georgia, Azerbaijan and Mozambique further confirms the identification of Genotype II and continuation of the post-Georgian continental jump of ASFV, suggesting a long-term geographical dissemination. The C-terminal region of the *B646L* gene which codes the major capsid protein p72 is known for its relatively high conservative nature and its sequencing is often the first approach in genotype assignment and epidemiological source tracking (Bastos et al., 2003; O’Donnell et al., 2024). Genotype I, although reported in the neighboring country of China, has not yet been detected in India (Ito et al., 2022). The 100% identity to other strains circulating in the country hints at possible epidemiological linkages and various means of dissemination have been suggested (Rotluangkimi et al., 2025; Parthiban et al., 2023). For further resolution, *E183L* (*p54*)-based phylogenetic tree was constructed which placed the current outbreak strains in Genotype IIa. Additionally, it was also observed that most of the *p54*-sequences submitted from India were also placed in IIa. The *p54*-based genotyping resulted in additional resolution of the molecular epidemiology of genotypes I, V, X and XX in an earlier study (Gallardo et al., 2009). However, whole genome sequencing, which was not performed in this study, provides greater resolution on the genomic and molecular epidemiology, including tracing the origin of the virus, and its absence limited us from drawing deeper molecular insights.

The data on the outbreaks provided by the state animal husbandry department indicated that the first outbreak was reported from Lamin, a village in West Jaintia Hills district of Meghalaya (WAHIS, 2025). As discussed earlier, Meghalaya is bordered by Assam on one side and Bangladesh on the other (**Figure 1**). During the period of first outbreak, *i.e.* July 2020; active cases were reported only from Assam and Arunachal Pradesh. The districts of Assam that were affected until July 2020 were Dibrugarh (January 2020), Dhemaji (February 2020), Biswanath (March 2020), Sivasagar (March 2020), Sonitpur (March 2020), Kamrup Metro (April 2020), Jorhat (April 2020), Majuli (May 2020), and Karbi Anglong (June 2020) (Patil et al., 2020; Buragohain et al., 2023; WAHIS, 2025). Although outbreaks were noticed in the Assam districts surrounding Meghalaya, Lamin is situated closer to the southern border of Meghalaya (**Figure 1** and **7**) nearer to Bangladesh, which reported its first case in 2023 (WAHIS, 2025). Additionally, the village is geographically disconnected from any waterways linking to the Brahmaputra River which was considered a route for the initial outbreaks in the country (Bora et al., 2020; Buragohain et al., 2023; Kimi et al., 2024). Though the village lies away from the major highway running through the northeast region *i.e*. NH6 which connects Assam, Meghalaya and Mizoram (Das et al., 2025b); it is located in close proximity to NH 206, a spur of NH6 running from Jowai in West Jaintia Hills to Mylliem in East Khasi Hills (Sarkar et al., 2024). It is well established that Meghalaya is deficient in pork production and imports animals from various parts of the country such as Assam, Haryana, Punjab, Karnataka, Rajasthan, Bihar, Uttar Pradesh and Mizoram (Kimi et al., 2024; Das et al., 2025a). NH6 usually forms the major conduit through which most pigs enter the state of Meghalaya in truck trailers through its northern borders (Das et al., 2025a). Cross border trade of pigs through the Mizoram-Myanmar border is also widely reported (Lalremruata et al., 2021; Kimi et al., 2024). Identification of the actual source of disease for the first outbreak remains intriguing and requires further investigation. Considering the circumstantial evidence, it can be assumed that the state was prone to transmission of ASF through movement of humans, animals or vehicles; as suggested earlier (Beato et al., 2022; Cheng and Ward, 2022; Gao et al., 2023; Galvis and Machado, 2024). This assumption is supported by outbreaks reported from Mawlyngkhung (Ri Bhoi) and Wapungskur (East Jaintia Hills) along NH6 in quick succession.

Ri Bhoi and East Khasi Hills were the most affected districts in terms of number of cases and number of outbreaks. As mentioned earlier, these districts form the main entry points for animals from Assam and NH6 run through them, which could have contributed to the dissemination of ASF. Although the government responded to the first outbreaks in the country by releasing the National Action Plan for control, containment and eradication of African Swine Fever in June 2020 (DAHD, 2020); limited time to act amidst COVID19 restrictions might have contributed to the occurrence of the first outbreak in the state. However, subsequent implementation of control measures as per the National Action Plan for control, containment and eradication of African Swine Fever such as culling and restriction on movement of animals might have resulted in temporary control of the disease at various time points and subsequent gradual reduction of ASF outbreaks in the state (Das et al., 2025a).

ASF, owing to its rapid spread, lack of vaccines and near 100% case fatality rate, often results in severe economic losses. Most of the tribal population of Meghalaya rear livestock mostly at a backyard scale, and there is often a lacuna in biosecurity measures considering the limitations in availability of labor and investment. The current study identified an economic loss of INR 2,74,45,883 (USD 305,225) arising from ASF outbreaks which occurred between 2020 up to April 2025. These values were indeed calculated with assumption that all animals that died would have reached slaughter age and been slaughtered. Few authors from India have already attempted to estimate the economic losses from ASF outbreaks based on various assumptions. Mohan et al. (2021) reported a loss of USD 39.9 million for 54,150 pigs from the Northeastern region of India. Hiremath et al. (2024) estimated the economic losses in four farms in Kerala resulting from an ASF outbreak and reported figures of around USD 12891 for 170 pigs. The differences may be due to the variations in assumptions and parameters used to arrive at the estimates. Since our study did not have data on the body weight of animals that died due to ASF, we resorted to estimation based on secondary data available from state government surveys. Moreover, our values report only the direct revenue loss arising from pork sales for each year. The estimate provided is therefore very conservative and should be extrapolated cautiously. A comprehensive study is required which also considers costs arising from disposal of carcass, diagnostics, therapeutics, control measures and revenue losses from trade; in addition to compensation paid to the farmers owing to culling of the animals. Additionally, ASF outbreaks necessitate restrictions on inter-state movement of animals, which also affect traders dependent on this trade for livelihood; thereby impacting all the stakeholders involved in pig and pork industry of the state as well as the country (Mohan et al., 2021; Das et al., 2025a).

Exploratory spatial data analysis through Moran’s *I* value, LISA and Getis-Ord-Gi* statistics indicated the signs of clustering of cases and outbreaks in 2020 and 2021. The hilly terrain of the state might have also contributed to the clustering of cases and outbreaks. The clustering pattern of the disease might have been disrupted in the following years by the rapid response measures aimed at containment of the disease. Additionally, we need to consider the diversity of breeds of animals, such as local, crossbreeds and exotic breeds, reared by the farmers in the region. Most of the farmers of Meghalaya rear animals at a backyard-setup with considerably small holding units of animals rather than large units which might have influenced the spatial dynamics (Milton et al., 2023b; Milton et al., 2024b). A descriptive spatio-temporal analysis in a wider north-east Indian region on the ASF outbreaks of 2020 and 2021 indicated low-low clustering and insignificant spatial clustering of ASF in districts of Meghalaya (Kimi et al., 2024). The discordance may be attributed to the difference in study area and spatial units.

Exploratory temporal data analysis revealed that no annual or seasonal trends were noticed in the outbreaks of ASF in Meghalaya, which was also supported by STL decomposition analysis. This aligns with previous reports, which did not identify any seasonal or annual trends in the occurrence of ASF (Schulz et al., 2019; EFSA, 2023). Seasonal trends, however, do exist in the transmission of ASF in wild boar populations in various regions (EFSA, 2017; Zakharova et al., 2023; Rogoll et al., 2023). These trends in wild boar populations may or may not align with those in domestic pigs (Rogoll et al., 2023; EFSA, 2023). Further studies are required to comprehensively compare the disease dynamics in domestic and wild pig populations in India. Additionally, internal variations in terms of altitude, rainfall and temperature range might have disrupted the seasonality pattern of the data, even if it was present. Moreover, the five-year study period might have been too short to exhibit observable annual or seasonal trends.

The role of wild animals and vectors was also considered while trying to understand the transmission dynamics of ASF in the state of Meghalaya. ASF has been reported in wild boar from Northeast India from Manas National Park, towards the northern border of Assam (Buragohain et al., 2023). ASF affection among wild boar population of Mizoram do exist as unofficial reports and confirmed genomic reports (Chingtham et al., 2024; Senthilkumar et al., 2025). Another study from India, reported the high mortality of free-ranging wild boars in forest areas of South India (Sai Balaji et al., 2024). The soft-tick vector of ASF, namely *Ornithodoros moubata*, has been reported in the eight north-eastern states (Govindarajan et al., 2023). No studies have documented their involvement of ticks in the transmission of ASF in India until now. With the increasing adverse effects of climate change, altered vector dynamics in terms of host-seeking behavior might play a major role in transmission of vector-borne disease and ASF is no exception (Bergmann et al., 2021; Tiwari et al., 2022);. Climate change also influences the feeding and movement behavior of wild animals in search of food along the domestic-wild interface. This is especially relevant in tribal areas, where no clearcut demarcation exists between the forest and inhabited areas. Additionally, swill feeding is a common practice prevalent in these geographical areas and might have contributed to the dissemination of the disease (Rotluangkimi et al., 2025).

Although we attempted to present a comprehensive record of the molecular epidemiological investigation, spatio-temporal trends of ASF outbreaks in Meghalaya with economic cost estimations, our study does have limitations that need to be considered when interpreting the results. One such limitation is the lack of precise data on all the outbreaks, including the age groups affected and body weight of the animals; which might have helped provide greater resolution for economic cost estimation. Another limitation is the smaller number of sample size and spatial units at the district level in Meghalaya and the absence of data from the surrounding areas, which reduces the power of spatial clustering analysis such as global Moran’s *I*, LISA and Getis-Ord-Gi* statistics. Although we have analyzed the available official data on confirmed ASF cases from state disease surveillance unit, the inferences drawn can only be considered exploratory. Moreover, the seropositive status of the animal population in the study area was not measured in the current study, which perhaps might have provided information about any possible subclinical or chronic circulation of the virus. The low mortality rates reported in this study may be attributed to hilly terrain and scattered settlements, which in turn leads to relatively low pig population density which limits the natural spread of the disease. Also, the figures reported are based only on ASF cases officially notified to the department through disease surveillance systems, so they tend to reflect only confirmed reports rather than the entire field situation. Additionally, control measures were put into action by the government department as per the National Action Plan for control, containment and eradication of African Swine Fever as laid down by Department of Animal Husbandry and Dairying (DAHD, 2020).

The study analyzed the long-term antigenic screening, molecular epidemiology and provided an exploratory spatio-temporal autocorrelation analysis on the ASF outbreaks occurring in the Northeast Indian state of Meghalaya over a period of five years. Understanding epidemiology and transmission is important for designing intervention strategies to contain the disease in the domestic pig population, which is reared by tribal people of Meghalaya, and forms an important part of their livelihood. The current study provided insights into the entry of the pathogen into the state and confirmed the circulation of Genotype II strains of ASF in Meghalaya. Further, the study provided an initial estimate of economic losses to the tribal stakeholders involved in pig farming. The spatial autocorrelation analyses indicated non-consistent clustering of cases throughout the year and weak but insignificant annual and seasonal periodicity of outbreaks in the state. Ri Bhoi and East Khasi Hills districts in Meghalaya played a major role in the ASF outbreaks and may be prioritized for targeted surveillance efforts with due consideration given to adequate testing on entry, biosecurity and quarantine measures.

## Data Availability

The original contributions presented in the study are included in the article/**Supplementary Material**. Further inquiries can be directed to the corresponding authors.

## Supplementary material

Supplementary Table 1 List of primers used in this study for diagnosis of ASF.

Supplementary Table 2 Details of district-wise first case/outbreak reported in a calendar year.

Supplementary Figure 1 LISA analysis of spatial data of aggregated and yearly ASF outbreak data.

Supplementary Figure 2 Getis-Ord-Gi* plots of aggregated and yearly ASF outbreak data.

Supplementary Figure 3 ACF and PACF plots of ASF outbreaks.
