# Supplementary material for: Molecular epidemiology, spatiotemporal patterns, and economic impact of African swine fever in Meghalaya, India
Source: Front Cell Infect Microbiol. 2026 Aug 12;16:1889985. doi: 10.3389/fcimb.2026.1889985 (PMC13506291; doi:10.3389/fcimb.2026.1889985)
Supplement: Supplementary file 1 [file Table1.docx]

**Supplementary Table 1:**

| **Primer** | **Oligonucleotides (5’ – 3’)** | **Target region** | **Amplicon**  **Size (bp)** | **Reference** |
| --- | --- | --- | --- | --- |
| ASF_p72_F | ATGGATACCGAGGGAATAGC | B646L | 278 | Wilkinson et al., 2000 |
| ASF_p72_R | CTTACCGATGAAAATGATAC |  |  |  |
| P72_UF | GGCACAAGTTCGGACATGT | B646L | 478 | Bastos et al., 2003 |
| P72_DR | GTACTGTAACGCAGCACAG |  |  |  |
| PPA722F | CGAAGTGCATGTAATAAACGTC | E183L | 683 | Gallardo et al., 2009 |
| PPA89 | TGTAATTTCATTGCGCCACAAC |  |  |  |

**REFERENCES**

Bastos, A.D., Penrith, M.L., Cruciere, C., Edrich, J.L., Hutchings, G., Roger, F., Couacy-Hymann, E.G.R.T. and R. Thomson, G., 2003. Genotyping field strains of African swine fever virus by partial p72 gene characterisation. *Archives of virology*, *148*(4), pp.693-706.

Gallardo, C., Mwaengo, D.M., Macharia, J.M., Arias, M., Taracha, E.A., Soler, A., Okoth, E., Martín, E., Kasiti, J. and Bishop, R.P., 2009. Enhanced discrimination of African swine fever virus isolates through nucleotide sequencing of the p54, p72, and pB602L (CVR) genes. *Virus genes*, *38*(1), pp.85-95.

Wilkinson PJ (2000) Manual of standards for diagnostic test and vaccines. In African swine fever. 4th edition. Paris, France: Office International Des Epizooties 189–198
