# Supplementary material for: Molecular epidemiology, spatiotemporal patterns, and economic impact of African swine fever in Meghalaya, India
Source: Front Cell Infect Microbiol. 2026 Aug 12;16:1889985. doi: 10.3389/fcimb.2026.1889985 (PMC13506291; doi:10.3389/fcimb.2026.1889985)
Supplement: Supplementary file 2 [file Table2.docx]

**Supplementary Table 2:**

| **District** | **Location** | **Date** |
| --- | --- | --- |
| **2020** | | |
| West Jaintia Hills | Lamin | 25-06-2020 |
| Ri Bhoi | Mawlyngkhung | 01-07-2020 |
| East Jaintia Hills | Wapungskur | 31-07-2020 |
| East Khasi Hills | Nongmynsong | 31-07-2020 |
|  | Mawshbuit Nongrim | 31-07-2020 |
| West Khasi Hills | Mairang | 03-08-2020 |
| South West Khasi Hills | Mawthong | 22-08-2020 |
| **2021** | | |
| Ri Bhoi | Syah Heh | 27-01-2021 |
| West Khasi Hills | Nongstoin | 02-01-2021 |
| South West Khasi Hills | Mawlangwir | 27-07-2021 |
| West Jaintia Hills | Nangbah | 14-09-2021 |
| East Khasi Hills | Nongkseh | 14-12-2021 |
| **2022** | | |
| Ri Bhoi | Umshorshor | 23-03-2022 |
| East Khasi Hills | Nongdawah | 21-06-2022 |
| **2023** | | |
| West Garo Hills | Dalu | 10-03-2023 |
|  | Asiragre | 10-03-2023 |
| Ri Bhoi | Umsawriang | 05-04-2023 |
| East Khasi Hills | Pynursla | 07-04-2023 |
| West Khasi Hills | Nongkasen | 07-04-2023 |
| South Garo Hills | Masighat | 14-04-2023 |
| **2024** | | |
| East Khasi Hills | Mawlyngbna | 29-02-2024 |
| Ri Bhoi | Nongsder | 13-03-2024 |
| South West Khasi Hills | Domjyrti | 13-05-2024 |
| West Khasi Hills | Nongshillong | 08-10-2024 |
| **2025** |  |  |
| East Khasi Hills | Mawdiangdiang | 03-01-2025 |
| West Jaintia Hills | Lad Japlem | 24-01-2025 |
